# Exercise and Episodic Specificity Induction on Episodic Memory Function

**DOI:** 10.3390/medicina55080422

**Published:** 2019-07-31

**Authors:** Paul D. Loprinzi, Kyle McRaney, Kathryn De Luca, Aysheka McDonald

**Affiliations:** Exercise & Memory Laboratory, Department of Health, Exercise Science and Recreation Management, The University of Mississippi, Oxford, MS 38677, USA

**Keywords:** cognition, physical activity, retrospective recall

## Abstract

*Background and objectives:* Episodic specific induction (ESI) is a manipulation shown to enhance episodic memory function. Episodic specificity induction involves thoroughly unpacking a recently encoded memory, with this enhanced retrieval-induced process helping to facilitate subsequent cognitions. In addition to ESI, emerging work suggests that acute exercise may also help to facilitate episodic memory function. The purpose of this study was to evaluate the potential individual and combined effects of acute exercise and ESI on subsequent episodic memory performance. *Materials and Methods:* Participants (*n* = 120) were randomly assigned into one of four groups, (1) ESI and exercise (ESI + E), (2) ESI only (ESI), (3) exercise only (E), and (4) no ESI and no exercise (Control; C). The ESI protocol involved watching a short video and then recalling details about the setting, people, and actions in the video. The exercise protocol involved an acute bout (15 min) of treadmill exercise. After these tasks, episodic memory function was evaluated with an autobiographical interview assessment and a computerized episodic memory task involving what–where–when integration. *Results:* We did not observe significant main effects for exercise or ESI on memory function but did observe some suggestive evidence of an interaction effect of these two parameters on episodic memory. That is, for the exercise group, memory performance was higher when combined with ESI as opposed to without ESI. *Conclusions:* Acute exercise and ESI may interact to influence episodic memory function.

## 1. Introduction

Episodic memory, part of the declarative memory system, involves the retrospective recall of information from a spatial–temporal context [1]. That is, episodic memory involves the integration of the what, where, and when aspects of a memory. Episodic specific induction (ESI) is a manipulation shown to enhance episodic memory function. Episodic specificity induction involves thoroughly unpacking a recently encoded memory, with this enhanced retrieval-induced process helping to facilitate subsequent cognitions. For example, ESI has been shown to enhance performance on subsequent tasks of episodic memory function [2,3], increase the generation of alternatives on future events [4], enhance imagination of future experiences [5], enhance creativity [6], and increase psychological well-being [7]. 

Our research group is interested in the effects of acute exercise on episodic memory function. Across several experiments, we have demonstrated that acute exercise may improve episodic memory performance [8,9,10,11,12,13]. We have observed these effects across multiple exercise intensities [8,9,10,11,12,13]. We have also discussed potential mechanisms of this effect, which likely involves increased neuronal excitability in several memory-related brain structures, such as the hippocampus [14,15,16,17].

Given these observations in the literature, it is conceivable that acute exercise and ESI may have an additive or interactive effect on enhancing episodic memory function. This question, however, has yet to be evaluated in the literature. Thus, the purpose of this study, written as a brief report, was to evaluate the potential individual and interactive effects of acute exercise and ESI on episodic memory function. We hypothesized there would be an interactive effect of acute exercise and ESI on episodic memory.

## 2. Methods

### 2.1. Study Design

A between-subject randomized controlled intervention was conducted. Participants were randomized into one of four conditions, including (1) ESI and exercise (ESI + E), (2) ESI only (ESI), (3) exercise only (E), and (4) no ESI and no exercise (Control; C). This study was approved by the ethics committee at the University of Mississippi (#19-066; approved on January 11, 2019). All participants provided written consent prior to participation. This study was not preregistered as a clinical trial. 

### 2.2. Participants

The study included 120 participants (*n* = 30 per group), none of whom dropped out of the study. Recruitment occurred via a convenience-based, non-probability sampling approach (classroom announcement and word of mouth). Participants included undergraduate and graduate students between the ages of 18 and 40 years.

Additionally, participants were excluded if they:

- self-reported as a daily smoker [18,19],
- self-reported being pregnant [20],
- exercised within five hours of testing [21],
- consumed caffeine within three hours of testing [22],
- had a concussion or head trauma within the past 30 days [23],
- took marijuana or other mind-altering drugs within the past 30 days [24], or
- were considered a daily alcohol user (>30 drinks/month for women; >60 drinks/month for men) [25]

### 2.3. Protocol for Visits

As stated, participants were randomized into one of four main protocols, including (1) ESI + E, (2) ESI, (3) E, and (4) C. The assessment protocol for these groups is shown in Table 1. Specific details regarding these assessments are discussed in the narrative following Table 1.

### 2.4. Exercise Assessment

Participants (ESI + E and E) engaged in moderate-intensity exercise on a treadmill for 15 minutes at 50% of their heart rate reserve [26]. Heart rate was monitored throughout the bout of exercise and the incline/speed was manipulated to keep the participant’s heart rate within five beats per minute of the target heart rate. 

### 2.5. Seated Rest Periods

During all seated rest periods, participants played an on-line version of Sudoku (easy difficulty level). This task was employed to prevent participant boredom. We have empirical work suggesting that this task (Sudoku) does not prime or enhance memory function and, thus, is a suitable task for control scenarios [27]. 

### 2.6. Episodic Specificity Induction (ESI)

Similar to other studies [28], the ESI involved participants watching a brief video (about an individual completing normal activities in the kitchen; referred herein as the “Tide Video”) and then recalling details about the setting, people, and actions in the video, using probes related to the surroundings, people, and actions from the video. Follow-up probes (open-ended questions) were used for each of these categories (ESI interview). Unlike the ESI condition, for the control condition, after watching the video, participants were asked to recall their general opinions, impressions, and thoughts about the video, followed by responding to several different questions from question banks related to the impressions of the environment, people, and actions (impressions interview). Thus, the main difference between the ESI and impressions control condition was that the ESI instructed participants to recall episodic details about the video, whereas the control condition instructed the participants to discuss their general impression of the video.

### 2.7. Memory Assessment

Two memory tasks were employed, in a fixed order, with the autobiographic interview occurring first, followed by the treasure hunt task. 

#### 2.7.1. Autobiographical Interview (AI)

The adapted AI (autobiographic interview) protocol, similar to other work [2], was used to assess the degree of internal and external detail generated from retrospective autobiographic memories. Participants viewed four different images that showed common scenes (e.g., library, park). For each image, participants had three minutes to complete the AI task. During this three-minute period, participants were asked to remember an actual event from the past few years, in as much detail as possible that is related to the picture. Responses were written down by the participant. After the three-minute period, participants rated, on a scale from 1 (not difficult at all) to 9 (extremely difficult), how difficult it was for them to come up with details from an actual event from their past (1, very easy; 3, easy; 5, neutral; 7, difficult; and 9, very difficult). 

Responses for the AI were scored based on internal and external details per image. Similar to other work [2], internal details for memory and imagination were any episodic and on-topic bits of information about the central event (e.g., people, objects, setting), whereas external details were semantic (e.g., facts and commentary), off-topic, or repetitive. Responses were scored by one of three coders, blind to the condition, who demonstrated adequate (ICC>0.80) inter-rater reliability for internal and external details on a packet of responses prior to the start of this experiment.

#### 2.7.2. Treasure Hunt Task (THT)

The THT (Treasure Hunt Task) is a computerized task assessing ‘what–where–when’ episodic memory, taking approximately 10 minutes to complete. Details of this THT have been discussed elsewhere [27,29,30]. In brief, this task involves ‘hiding’ items in various scenes, then later indicating what items were hidden, where, and on what occasion. This requires the integration of item, location, and temporal memory into a single coherent representation (what–where–when memory, WWW). Internal consistency for this measure has been previously demonstrated (ICCs > 0.7) [30]. This study used the ‘hard’ difficulty version of the task, assessing 24 unique item–location–time combinations. The outcome measure for this task was the correct proportion (out of 100%) of the integration of item, location, and temporal memory (i.e., WWW). 

### 2.8. Statistical Analyses

All statistical analyses were computed in JASP (v. 0.9.2). For the AI outcome, a two-factor mixed-measures ANOVA was conducted. Specifically, a 2 (ESI vs. E) × 2 (internal details vs. external details) design was employed. The between-subject factor involved whether the participant received the ESI induction (coded as 1 or 0) and/or an exercise manipulation (coded as 1 or 0). The within-subject factor involved the internal details (sum of responses from the four internal details assessments) and external details (sum of responses from the four external details assessments). Thus, the 2 × 2 RM-ANOVA involved two levels of the between-subject factor (ESI, exercise) and two levels of the within-subject factor (internal details, external details). In this 2 × 2 RM-ANOVA, in addition to evaluating a main effect for ESI and a main effect for exercise, we evaluated whether there was an ESI by exercise interaction. For the THT outcome, a two-factor independent groups ANOVA was conducted. The outcome variable was the proportion correct on the THT task and the two main effects were ESI and exercise. In addition to these two main effects, we evaluated whether there was an ESI by exercise interaction.

## 3. Results

Table 2 displays the demographic characteristics of the sample across the four independent groups. As noted in Table 2, there were no statistically significant differences in the demographic and behavioral parameters across the four experimental groups. 

Table 3 displays the physiological responses (heart rate) to the experimental manipulations. As expected, we observed a statistically significant time (*p* < 0.001), group (*p* < 0.001), and time by group interaction (*p* < 0.001). The non-exercise groups maintained a heart rate in the low- to mid-70s, whereas the heart rate increased up to 130–140 beats per minute during the exercise session. 

Table 4 displays the memory scores across the experimental conditions. For the AI assessment, there were no statistically significant differences in the perceived difficulty level of coming up with past memories (*p* = 0.57). For the AI outcome, there was a statistically significant main effect for details (*p* < 0.001), in that participants recalled more external details than internal details (*M*_diff_ = 20.3, SE = 0.82, *t* = 24.6, *p* < 0.001). There was no statistically significant main effect for exercise (*p* = 0.09) and no details by ESI by exercise interaction (*p* = 0.64). 

**Figure 1:**
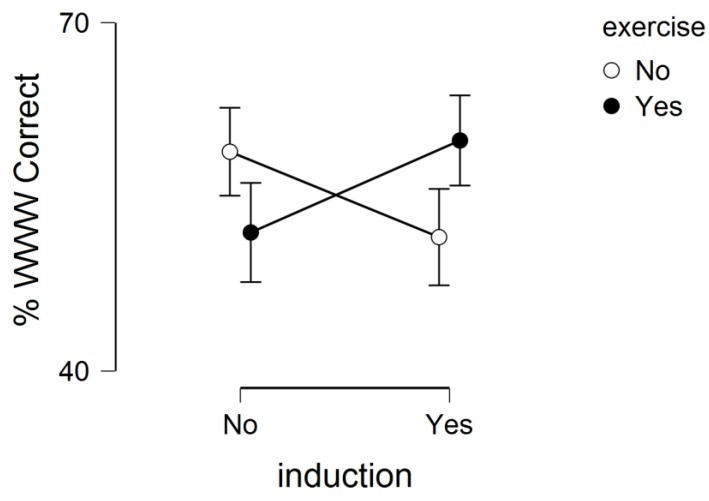
Interaction effect of exercise and ESI on overall memory performance. Error bars represent standard error bars.

For the THT, there was no main effect for ESI (*p* = 0.94) or exercise (*p* = 0.86), but there was a marginally statistically significant ESI by exercise interaction (*p* = 0.06). This interaction effect is shown in Figure 1.

## 4. Discussion

The present experiment, written as a brief report, evaluated the individual and combined effects of acute exercise and ESI on episodic memory function. In this experiment, we did not observe a statistically significant main effect for ESI on episodic memory function. That is, engaging in an ESI session, which involved unpacking a recently encoded memory (Tide Video), did not help facilitate memory retrieval on a subsequent memory (AI or THT). Although there was a marginally (*p* = 0.09) significant main effect for exercise, it did not reach statistical significance, suggesting that acute exercise did not statistically alter memory performance (AI or THT). We did, however, observe some evidence of a potential interaction effect of acute exercise and ESI on THT performance (*p* = 0.06). That is, for the exercise group, THT memory performance was higher when combined with ESI as opposed to without ESI. This suggests a potential interaction effect of acute exercise and ESI on overall episodic memory function. This finding, however, should be interpreted with caution, as this interaction effect may be, partially, influenced by the non-expected difference in WWW memory performance in the non-exercise conditions across the ESI conditions. That is, WWW memory performance was higher in the non-exercise conditions when ESI was not present.

An interesting observation of our study was the suggestive evidence of a potential interaction effect of acute exercise and ESI on episodic memory function. Acute exercise, by itself, was not associated with episodic memory, but when coupled with ESI, there was suggestive evidence of an interaction effect. This explanation may, in part, be influenced by the intensity level of the acute bout of exercise. Our previous work suggests that high-intensity acute exercise (vs. moderate intensity, which was employed in the present experiment) is more beneficial in enhancing episodic memory function [31,32]. We intentionally chose to employ a moderate-intensity acute exercise protocol for two reasons. First, although high-intensity exercise (vs. moderate intensity) may favor episodic memory performance, past research has shown that acute moderate-intensity exercise may also improve episodic memory [13]. Second, the general population is likely to have a more favorable exercise-induced affective response during moderate-intensity (vs. vigorous-intensity) exercise, and as such, to maximize generalizability of our findings to the broader population, we elected to employ a moderate-intensity protocol. It is possible that our moderate-intensity exercise protocol, by itself, was not sufficient to enhance episodic memory, but when coupled with ESI, memory performance was higher. 

As an extension of this study, future work should consider employing a within-subject design, as opposed to our employed between-subject design. It is likely that a within-subject design would have allowed for greater statistical power to observe statistically significant main effects. Future work should also consider implementing a higher-intensity bout of acute exercise, as higher-intensity acute exercise may have a greater effect on long-term potentiation, a cellular mechanistic correlate of episodic memory. Future work on this topic should evaluate candidate mechanisms of this paradigm. It is also possible that the exercise duration (e.g., 15 min) employed herein was not a sufficient stimulus, and as such, future work may wish to consider longer durations of acute exercise. Future work should also continue to evaluate multiple memory types and assessments. In our present experiment, we demonstrated some evidence of an interaction effect for acute exercise and ESI on THT, but not on AI. Although speculative, perhaps this was influenced by the difficulty level of the memory tasks. For example, for the AI task, after each image, participants rated how difficult it was for them to come up with an actual event from the image. The mean image rating (Table 4) was close to 3, which corresponds to “easy” (1, very easy; 3, easy; 5, neutral; 7, difficult; and 9, very difficult). However, for the THT task, we employed a high-difficulty level task, involving 24 unique item–location–time combinations.

## 5. Conclusions

In conclusion, in this experiment, we did not observed evidence of a main effect for acute exercise or ESI on episodic memory function. However, we observed some suggestive evidence of a potential interaction effect of acute exercise and ESI on episodic memory.

## Figures and Tables

**Table 1:** Study Protocol.

Condition	Start	→	→	→	→	Finish	
ESI + E	15 min exercise	5 min rest	Tide video	Math for 3 min	ESI interview	AI	THT
ESI	20 min rest		Tide video	Math for 3 min	ESI interview	AI	THT
E	15 min exercise	5 min rest	Tide video	Math for 3 min	Impressions interview	AI	THT
C	20 min rest		Tide video	Math for 3 min	Impressions interview	AI	THT

AI, autobiographical interview; C, control; ESI, episodic specificity induction; E, exercise; THT, treasure hunt task.

**Table 2:** Characteristics of the sample.

	ESI + E	ESI	E	C	*p* Value
*n*	30	30	30	30	
Age, mean years	21.3 (1.1)	21.4 (1.5)	21.1 (1.2)	21.0 (1.5)	0.69
Gender, % female	70.0	80.0	73.3	80.0	0.75
Race-ethnicity, % white	63.3	43.3	40.0	60.0	0.40
BMI, mean kg/m^2^	26.4 (4.6)	27.1 (6.1)	28.6 (7.5)	26.1 (5.2)	0.36
MVPA, mean min/week	174.4 (119.7)	125.3 (114.8)	121.0 (113.5)	150.0 (138.6)	0.33

BMI, body mass index; MVPA, moderate-to-vigorous physical activity (self-reported). Values in parentheses are standard deviations.

**Table 3:** Physiological (heart rate) responses to the experimental manipulations.

Heart Rate	ESI + E	ESI	E	C	*p* Value
Baseline	75.2 (11.0)	79.8 (13.9)	78.1 (12.7)	78.3 (12.8)	F(time) = 503.2, *p* < 0.001, η^2^_p_ = 0.81
Midpoint	131.5 (11.5)	82.8 (14.8)	131.0 (13.6)	73.2 (12.3)	F(group) = 124.0, *p* < 0.001, η^2^_p_ = 0.76
Endpoint	138.0 (7.2)	83.0 (17.1)	140.1 (9.8)	72.9 (11.2)	F(time × group) = 182.7, *p* < 0.001, η^2^_p_ = 0.83

A 3 (timepoint) × 4 (condition) RM-ANOVA was conducted to calculate *p* values and effect sizes (η^2^_p_). Values in parentheses represent standard deviations.

**Table 4:** Memory outcomes across the experimental conditions.

Memory Type	ESI + E	ESI	E	C	*p* Value
AI					
Internal Details	3.7 (4.8)	3.1 (3.5)	3.0 (3.5)	2.9 (3.3)	Details: F(1,116) = 605.1, *p* < 0.001, η^2^_p_ = 0.84 ESI: F(1,116) = 1.24, *p* = 0.26, η^2^_p_ = 0.01 Exercise: F(1,116) = 2.86, *p* = 0.09, η^2^_p_ = 0.02 ESI × Exercise: F(1,116) = 0.43, *p* = 0.50, η^2^_p_ = 0.004 Details × ESI: F(1,116) = 0.44, *p* = 0.50, η^2^_p_ = 0.004 Details × Exercise: F(1,116) = 2.34, *p* = 0.12, η^2^_p_ = 0.02 Details × ESI × Exercise: F(1,116) = 0.21, *p* = 0.64, η^2^_p_ = 0.002
External Details	26.3 (11.3)	22.4 (7.0)	23.6 (8.5)	21.8 (8.4)	
Difficulty Level	3.4 (1.4)	3.2 (1.1)	3.2 (1.1)	2.9 (1.3)	ESI: F(1,116) = 1.21, *p* = 0.27, η^2^_p_ = 0.01 Exercise: F(1,116) = 1.46, *p* = 0.22, η^2^_p_ = 0.01 ESI × Exercise: F(1,116) = 0.31, *p* = 0.57, η^2^_p_ = 0.003
THT					
% Correct	59.9 (21.2)	51.5 (22.7)	51.9 (23.3)	58.9 (20.7)	ESI: F(1,116) = 0.005, *p* = 0.94, η^2^_p_ = 0.001 Exercise: F(1,116) = 0.03, *p* = 0.86, η^2^_p_ = 0.001 ESI × Exercise: F(1,116) = 3.60, *p* = 0.06, η^2^_p_ = 0.03

For the AI outcome, a two-factor mixed-measures ANOVA was conducted. For the THT outcome, a two-factor independent groups ANOVA was conducted. Values in parentheses represent standard deviations. AI, autobiographical interview, THT, treasure hunt task.
